# Transplantation of bone marrow mesenchymal stem cells improves cognitive deficits and alleviates neuropathology in animal models of Alzheimer’s disease: a meta-analytic review on potential mechanisms

**DOI:** 10.1186/s40035-020-00199-x

**Published:** 2020-05-27

**Authors:** Chuan Qin, Yalan Lu, Kewei Wang, Lin Bai, Guiying Shi, Yiying Huang, Yongning Li

**Affiliations:** 1 Institute of Laboratory Animal Sciences, Chinese Academy of Medical Sciences & Comparative Medical Center, Peking Union Medical College, Beijing Engineering Research Center for Experimental Animal Models of Human Critical Diseases, 5 Panjiayuan Nanli St, Beijing, 100021 China; 2 Department of International Medical Service & Department of Neurosurgery, Peking Union Medical College Hospital, Chinese Academy of Medical Sciences and Peking Union Medical College, Shuaifuyuan 1, Dong Cheng District, Beijing, 100730 China

**Keywords:** Alzheimer’s disease, Bone marrow mesenchymal stem cells, Meta-analysis, Amyloid β peptide, Memory loss, Cognitive deficits, Animal model, Neuropathology

## Abstract

**Background:**

Alzheimer’s disease is a neurodegenerative disorder. Therapeutically, a transplantation of bone marrow mesenchymal stem cells (BMMSCs) can play a beneficial role in animal models of Alzheimer’s disease. However, the relevant mechanism remains to be fully elucidated.

**Main body:**

Subsequent to the transplantation of BMMSCs, memory loss and cognitive impairment were significantly improved in animal models with Alzheimer’s disease (AD). Potential mechanisms involved neurogenesis, apoptosis, angiogenesis, inflammation, immunomodulation, etc. The above mechanisms might play different roles at certain stages. It was revealed that the transplantation of BMMSCs could alter some gene levels. Moreover, the differential expression of representative genes was responsible for neuropathological phenotypes in Alzheimer’s disease, which could be used to construct gene-specific patterns.

**Conclusions:**

Multiple signal pathways involve therapeutic mechanisms by which the transplantation of BMMSCs improves cognitive and behavioral deficits in AD models. Gene expression profile can be utilized to establish statistical regression model for the evaluation of therapeutic effect. The transplantation of autologous BMMSCs maybe a prospective therapy for patients with Alzheimer’s disease.

## Background

Alzheimer’s disease (AD) is a chronic disorder of central nervous system. Its clinical manifestations are characterized by memory loss, cognitive dysfunction, abnormal behavior, etc. With the deterioration of AD, patients fall into stupor state and usually die of exhaustion within 5—10 years [1].

In pathology, AD is manifested by the decreased number of neurons and synapses in cerebral regions, resulted in different degrees of memory loss and cognitive impairment. Amyloidal plaques, mostly insoluble deposits of amyloid β peptide (Aβ), are observed around neurons [1]. Neurofibrillary tangles, hyperphosphorylated aggregates of the microtubule-associated protein tau, are accumulated inside the neuron [2]. As compared with the general aging brain, many plaques and tangles in patients with AD are discovered in specific brain regions such as temporal lobe and hippocampus [3, 4].

Currently, there is no cure for the Alzheimer’s disease. Therapeutic strategy in the treatment of AD is to alleviate symptoms through pharmacological intervention, such as an enhancement of neurotransmitter acetylcholine [5, 6]. A few medicines can slow down the exacerbation and improve behavioral deficits in some patients. Two types of medication are presently used to treat cognitive symptoms, including (i) cholinesterase inhibitors (AChE inhibitors) such as donepezil, galantamine and rivastigmine [6]. These drugs boost levels of acetylcholine that is decreased in the brain of Alzheimer’s disease, which may improve neuropsychiatric agitation or depression; (ii) memantine, an uncompetitive NMDA antagonist, is used to improve memory and awareness in moderate to severe patients with AD. It works in cell communication network and delays the exacerbation of symptoms due to AD. Sometimes, the memantine is utilized in combination with AChE inhibitors. Antidepressants may be prescribed to control the behavioral symptoms associated with Alzheimer’s disease. The therapeutic effect of above drugs is limited in advanced patients with poor condition. Recent nanotechnological advancements provide effective options of drug carriers [7, 8]. For instance, when the rivastigmine was assisted with biocompatible nanoparticles (NPs), the NPs-based drug delivery could effectively cross the blood-brain barrier and improved its bioavailability [7]. The biocompatible NPs also showed significant effect on the kinetics of Aβ-fibrinogen [9, 10]. In addition, non-pharmacological approaches such as diet, regular exercise or other healthy lifestyle choice are supplemented for the improvement of patients’ life quality.

Transplantation of mesenchymal stem cells (MSCs) as a therapeutic technique has been well developed in the recent decades. It has also been explored in the treatment of animal models with nervous disease. The accumulative evidence demonstrated that the transplanted MSCs could be differentiated into cell lineage such as neurons and reconnected synaptic network, which played a critical role in the functional improvement of nervous system [11, 12]. A comparison had been carried out among stem cells derived from different resources such as brain, fat, bone marrow, umbilical blood or fetal tissues [13–15]. Owing to ethical issue and alloimmunogenicity, stem cells from embryos and allogenic umbilical cord may be not suitable for the treatment of AD. Autologous neurons from brain biopsy are confronted with unacceptable attitude and challenge. Still, it is long way to go for the preparation of stem cells through iPSc method. Therefore, autologous stem cells from bone marrow or fat were additional choices. Interestingly, the stem cells from bone marrow had a better therapeutic effect as compared with that from the fatty tissue based on previous studies [16, 17]. The MSCs from autologous bone marrow could be delivered into AD subjects via different approaches such as intracerebral, peripheral vein and intracerebroventricular injection. The therapeutic effect of bone marrow mesenchymal stem cells (BMMSCs) was verified in several animal models. The results indicated that the BMMSCs could alleviate the memory loss, behavioral deficits and neuropathology. Technical advantages in autologous BMMSCs have provided a prospective therapy for patients with Alzheimer’s disease.

The early studies demonstrated that the therapeutic effect of exogenous stem cells could improve pathological manifestations in animal models with Alzheimer’s disease. Furthermore, there were seldom adverse responses following a transplantation of bone marrow mesenchymal stem cells [17, 18]. Advantages of bone marrow mesenchymal stem cells were reflected by its efficiency and safety. At present, the transplantation of bone marrow mesenchymal stem cell has been optimized through appropriate facilities as well as experimental conditions. A series of research data proved a dramatic improvement in cognitive deterioration and neuropathological symptoms among AD-like animal models. Autologous bone marrow-derived mesenchymal stem cells may be used in the clinical treatment of Alzheimer’s disease in near future. The present study aims to explore the potential mechanisms by which the transplantation of BMMSCs improves cognitive and behavioral deficits in animal models of Alzheimer’s disease, which can lay a foundation for the clinical application of autologous BMMSCs in AD patients.

## Methods

### Systematical search of published literature

Database PubMed, Medline, and Embase were systematically screened, and the time point was set at the end of February 2019. Keywords “Alzheimer’s disease” and “stem cell transplantation” were used to identify literature. Total 414 references were acquired, which were not restricted by the type of publication. The published work was further scrutinized according to the integrity of data and article types.

### Study selection

Studies eligible for inclusion were based on quality of resultant data, included randomized controlled trials and cohort-controlled trials. We excluded studies using therapeutic stem cells from umbilicus cord, fat and brain. Also, the exclusion covered studies that provided incomplete data relevant to the pre-specified outcome variables. The inclusion studies were restricted to the transplantation of BMMSCs. Data extraction was accomplished by two investigators independently.

### Data collection and outcome measures

The extracted data were based on general characteristics of all included studies, such as source of reference, study design, animal species, surgery procedure, delivery route of stem cells, outcome measures, etc. A primary comparison was performed among primary data derived from BMMSCs and control groups. The data analysis involved cognitive function, behavioral change, neurogenesis, angiogenesis, apoptosis, inflammatory response, immunomodulation, reactive gliosis, microglial activation, level of Aβ peptide, tau hyperphosphorylation and so forth. Morbidity of adverse response was calculated by the number of animals with at least one complication after stem cell transplantation and mortality was computed by death number during or after operation due to any causes. Meta-analysis based on outcome variables was further carried out, including Y-maze test, escape latency, histone H3-positive cells, expression of VEGF, TNF-α, IL-1β, Aβ level, activation of Aβ-degrading enzyme ECE, percentage of Iba-1 positive cells, percentage of AT8 positive cells, etc.

### Statistical analysis

Review Manager (RevMan version: 5.3.5; Copenhagen: The Nordic Cochrane Centre, The Cochrane Collaboration, 2014) was used to pool data and meta-analysis. For categorical variable, treatment effect was expressed as odds ratio (OR) with corresponding 95% confidence intervals (CI). Results were compared through a random-effects model. For continuous variable, treatment effect was expressed as weighted mean difference (WMD) with corresponding 95% CI. Chi-square (Chi^2^ or χ^2^) and I^2^ statistics estimate the appropriateness of pooling individual study. Heterogeneity was evaluated by χ^2^-test with significance set at *P* value 0.10. The heterogeneity was measured by I^2^ more than 50% as statistical significance. Forest plots were constructed, *P* values of < 0.05 as significant difference. Gene data on microarray and high-throughput DNA sequencing were retrieved out of Geo DataSets (https://www.ncbi.nlm.nih.gov/pubmed/). The linear relationship between the two variables was measured with Pearson’s correlation coefficient. Principal component analysis (PCA) of gene expression data was performed based on the correlation matrix. The number of principal components would satisfy more than 80% variability of differential gene expression. The clusters were combined based on similar expression profiles and enriched gene ontology (GO) categories. The cluster analysis was performed using correlation for hierarchical clustering and Euclidean distance for K-means clustering. Difference was considered significant at *p* values < 0.05. Data were analyzed with software SPSS 19.0 (IBM Corp., Armonk, NY, USA), JMP 13.0 software (SAS Institute Inc., Cary, North Carolina, USA), and R 3.5.3 for Windows.

## Main text

### Quality assessment of the included studies

**Fig. 1:**
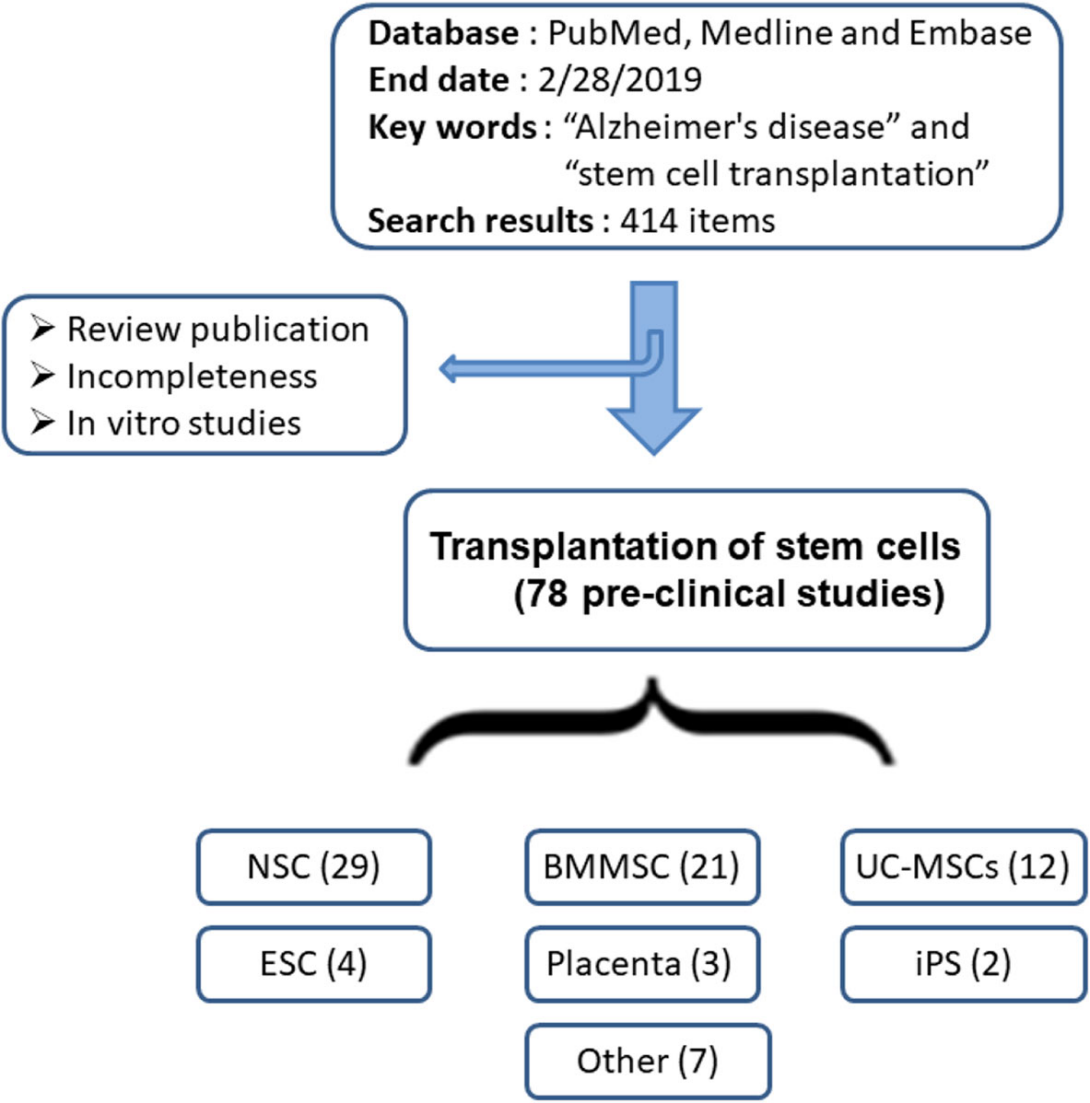
Transplantation of bone marrow mesenchymal stem cells could improve clinical manifestations in animal models with Alzheimer’s disease. Flow chart summarized relevant references that was identified and included in the meta-analytic review

**Fig. 2:**
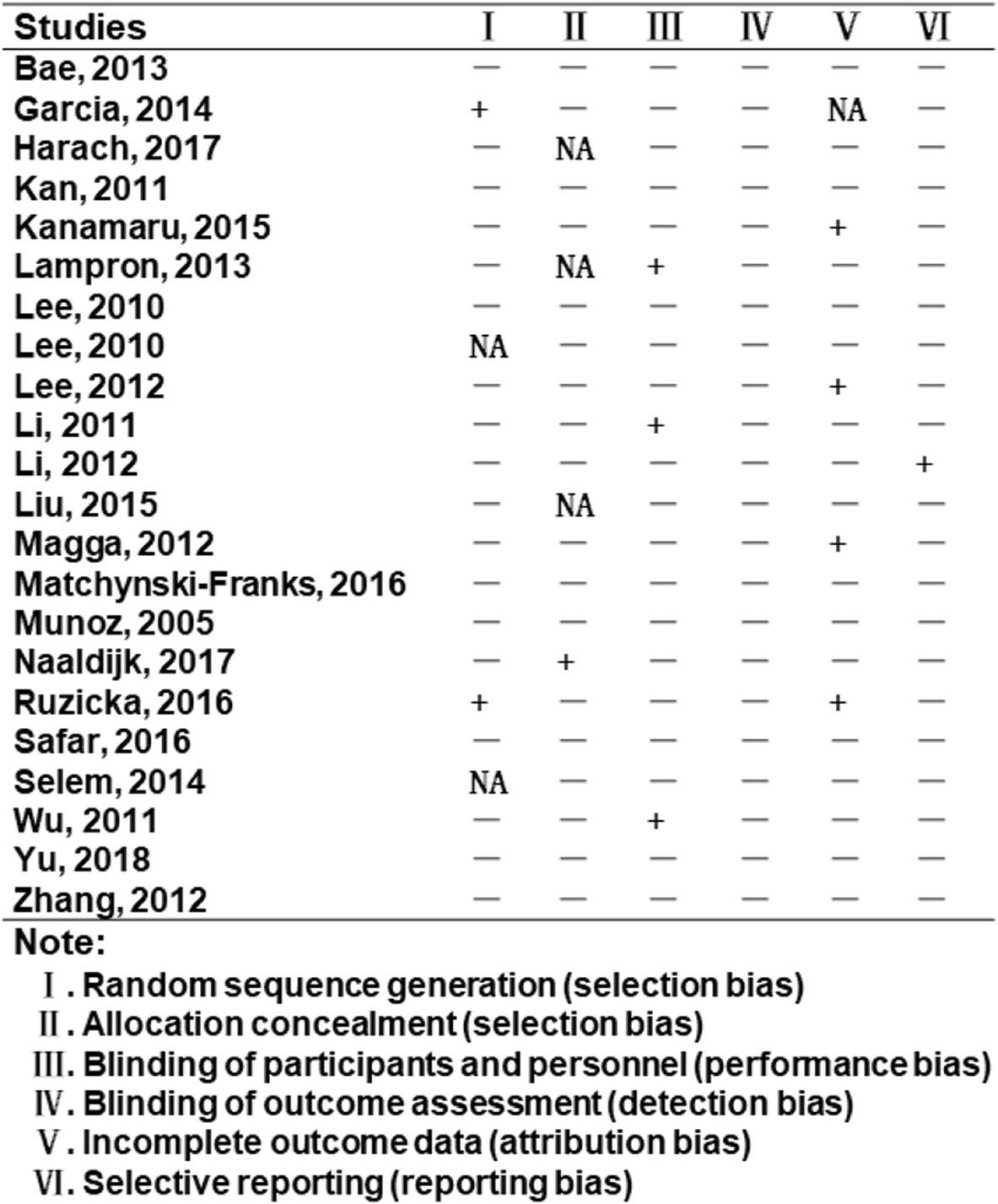
Summary of potential bias in the identified studies

Systematic review on therapeutic effect of mesenchymal stem cells for Alzheimer’s disease was summarized according to animal species, sources of mesenchymal stem cells, cognitive improvement, route of delivery, position of delivery, mechanisms, and so on (Supplementary table). Original studies with complete data were kept in the present meta-analytic review (Fig. 1, Table 1). General characteristics of the included studies in the meta-analysis were reflected by source of transplanted stem cells, amount of transplanted stem cells, species of recipient animals, gender ratio of recipients, age or body weight of recipients, route of delivery, position of delivery, and sustainability of transplanted stem cells (Table 2). Study quality was assessed via bias in primary studies. Potential bias in the identified studies were also evaluated (Fig. 2). The interpretation of results was weighed in terms of existed bias and sources of heterogeneity. The methodology of included studies was evaluated through random sequence generation, blinding of outcome assessors, incomplete outcome data, and selective reporting, etc. Priori criteria of high-quality study include (i) randomized trial; (ii) controlled study; (iii) adequately reported methodology of measurement.

**Table 1:** Transplantation of BMMSCs for the treatment of animal models with Alzheimer’s disease. Keywords “Alzheimer’s disease” and “stem cell transplantation” were utilized to screen database PubMed, Medline, and Embase respectively. Studies eligible for inclusion were restricted to the bone marrow mesenchymal stem cells. Primary studies with complete data were retained in the current meta-analytic review

Studies	Study design	Results	Mechanisms	References
Bae, 2013	Random APP/PS1 mice, in vivo study.	Decline of amyloid-beta deposits and and the improvement of synaptic transmission	Significant decrease in the cerebral Aβ deposition; Expression of dynamin 1 and Synapsin 1, key pre-synaptic proteins.	Curr Alzheimer Res. 2013 Jun;10 [5]:524–31
Garcia, 2014	Random 2xTg-AD male congenic mice, in vivo study. BMMSCs over expressed VEGF (human VEGF 165 cDNA from uP-VEGF)	Behavioral benefits included the recovery of memory loss and cognitive deficits as demonstrated by open-field evaluation, social recognition test, and plus-maze discriminative avoidance task (PM-DAT).	Mechanisms involved neovascularization, reduction of amyloid-beta plaques, and to decrease astrocytes and microglial cells	Front Aging Neurosci. 2014 Mar 7;6:30
Harach, 2017	Random APP/PS1 mice, in vivo study. Stem cells were obtained from Stemedica Cell Technologies (SanDiego, USA). The cells are equivalent to commercially available stem cells from ThermoFisher Scientific “StemPro BM MSC” (part number A15653)(ischemia-tolerant mesenchymal stem cells)	Significant reduction of cerebral Ab plaques and neuroinflammation	Reduced cerebral Aβ plaques,increasing NPE,IDE and ECE Aβ-degrading enzymes;reduced TNFa,IL-12p70 and IL-10.	Neurobiol Aging. 2017 Mar;51:83–96.
Kanamaru, 2015	Random APP/DAL101 mice,in vivo study. To confirm preventive effect of BMMSCs against neuronal degenerationr or therapeutic effect of BMMCs on neuronal degeneration respectively.	To suppress neuronal loss and restore memory impairment of DAL mice,to reduce Aβdeposition and improve cognitive behavior in APP mice.	To prevent neurodegeneration and Aβ deposition.	Brain Res. 2015 Apr 24;1605:49–58.
Lampron, 2013	Random APP/PS1 mice, in vivo study.	Bone marrow-derived cells (BMDC) under stimulaton of M-CSF could infiltrate the CNS in animal models for stroke and Alzheimer’s disease. They were confined in diseased sites for several weeks.	Hypoxic-ischemic injury sites or amyloid plaques could induce the entry of BMDC cells.	J Comp Neurol. 2013 Dec 1;521 [17]:3863–76.
Lee, 2010	Random C57BL/6 mice were injected with aggregated Aβto make AD model, in vivo study. The bone marrow cells were cultured for 1 week, and the plastic-adherent population was used for subsequent experiments.	To attenuate memory impairment and to inhibit neuronal apoptosis.	To reduce aβ deposition, stimulate microglial activation, switch the microglial phenotype into alternative form, decrease tau hyperphosphorylation, and diminish Aβ-induced oxidative stress in model animals.	Curr Alzheimer Res. 2010 Sep;7 [6]:540–8
Lee, 2010	Random APP/PS1 mice. The bone marrow cells were cultured for 1 week, and the plastic-adherent population was used for subsequent experiments.	To ameliorate Abeta-induced neuropathology and improve the cognitive decline associated with Abeta deposits.	To modulate immune/inflammatory responses and to restore defective microglial functionin AD mice, as evidenced by increased Abeta-degrading factors, decreased inflammatory responses, elevation of alternatively activated microglial markers, and diminished tau hyperphosphorylation.	Stem Cells. 2010 Feb;28 [2]:329–43.
Lee, 2012	Random APP/PS1-GFP chimeric mice,in vivo study;Therapeutic effect;	Alternative microglia activation to eliminate Abeta deposition in the AD brain, and further improve behavior.	The icroglial activation and migration into the brains of Abeta-deposited AD mice via elevation of the chemoattractive factor, CCL5.Neprilysin and interleukin-4 derived from the alternative microglia were associated with a reduction in Abeta deposition and memory impairment in AD mice.	Stem Cells. 2012 Jul;30 [7]:1544–55.
Li, 2011	Random APP/PS1 mice,mechanistic study.	Systemic administration of SCF + G-CSF reduced beta-amyloid deposition in AD mice, and increased the number of bone marrow-derived microglial cells in the brain.	Decreased β-amyloid deposition, enhanced microglial	Alzheimers Res Ther. 2011 Mar 15;3 [2]:8
Li, 2012	Random rat experiments,in vivo study; Therapeutic effect;	To improve spatial learning and memory ability as demonstrated by Morris water maze experiment	BM-MSCc could migrate through the blood-brain barrier and survived in the hippocampus of AD rats	Zhejiang Da Xue Xue Bao Yi Xue Ban. 2012 Nov;41 [6]:659–64
Liu, 2015	Random APP/PS1 mice; Overexpression of as-miR-937 in MSCs may improve the therapeutic effects of MSCs on AD	MSCs reduced the deposition of amyloid-beta peptide aggregates (Aβ) and improved behavior as proved by social recognition test (SR) and plus-maze discriminative avoidance task (PM-DAT).	MSCs significantly increased Brn-4 protein levels, which reduced the deposition of Aβand upregulated the levels of BDNF in AD mice.	Cell Physiol Biochem. 2015;37 [1]:321–30
Magga, 2012	Transgenic APdE9 mice, BM-derived haematopoietic stem cells (HSC)	HSC-derived monocytic cells (HSCM) could be genetically modified and contributed to Abeta reduction in APdE9 mouse model of AD .	HSC-derived monocytic cells (HSCM) uptook Abeta protein and reduced Aβburden in AD mouse brain.	J Cell Mol Med. 2012 May;16 [5]:1060–73
Matchynski-Franks, 2016	Random 5xFAD mice; the optimal location for transplanting MSCs; Injection into the lateral ventricles was better than the injection into hippocampus.	MSC transplants effectively reduced learning deficits in the 5xFAD mouse model as demonstrated by radial-arm water maze 8-choice memory task, water t-maze two-choice learning task, spontaneous motor activity, motor coordination, and prepulse inhibition.	Significantly to decrease the level of Abeta42 in the brains of 5xFAD mice subsequent to transplantation of MSCs.	Cell Transplant. 2016;25 [4]:687–703.
Naaldijk, 2017	Random APP/PS1 mice,in vivo study. Therapeutic effect of BMMCs	MSCs may affect AD pathology (neuroinflammation) via an immune-modulatory function that includes an effect on microglial cells.	To reduce the expressional levels of TNF-alpha, IL-6, MCP-1, and NGF in MSC recipients. Also,to reduce the size of pE3-Abeta plaques in the hippocampus.	Neuropathol Appl Neurobiol. 2017 Jun;43 [4]:299–314.
Ruzicka, 2016	Random 3xTg-AD mice treated by human MSCs. Therapeutic effect of BMMCs	Learning Deficits improved; reduced Amyloid β (Aβ*56); increased neurogenesis;	Clusters of proliferating cells in the subventricular zone; the level of glutamine synthetase; downregulation of Abeta*56 levels in the entorhinal cortex	Int J Mol Sci. 2016 Jan 25;17 [2]. pii: E152.
Safar, 2016	Adult male Wistar rats, effects of bone marrow-derived (BM) EPCs transplantation,endothelial progenitor cells (EPCs)	Improved the learning and memory deficits, and mitigated the deposition of amyloid plaques and downregulation of p-tau. To correct memory deficits and AD-like pathological dysfunction	Downregulation of p-tau and its upstream glycogen synthase kinase-3beta (GSK-3beta); corrected the perturbations of neurotransmitter levels including acetylcholine, dopamine, GABA, and the neuroexitatory glutamate; to boost the expression of vascular endothelial growth factor (VEGF), nerve growth factor (NGF), brain-derived neurotrophic factor (BDNF) and its upstream cAMP response element binding (CREB); suppression of the proinflammatory tumor necrosis factor-alpha (TNF-alpha), interleukin-1beta (IL-1beta); upregulation of interleukin-10 (IL-10), Nrf2 and seladin-1.	Mol Neurobiol. 2016 Apr;53 [3]:1403–1418
Selem, 2014	Adult female Sprague–Dawley rats,,in vivo study. Therapeutic effect of BMMCs	To remove beta-amyloid plaques from hippocampus; anti-apoptotic, neurogenic and immunomodulatory properties	Proliferating the number of positive cells for choline acetyltransferase (ChAT) and survivin expression, as well as selective AD indicator-1 (seladin-1) and nestin gene expression. Histopathological examination indicated the removal of beta-amyloid plaques from hippocampus. Significant improvement in these biomarkers was similar to or better sometimes than the reference drugs.	Cell Biol Int. 2014 Dec;38 [12]:1367–83
Wu, 2011	Random SD rat experiments via hippocampal fimbria-farnix (FF) amputation model, Ginsenoside Rg1 treatment,in vivo study. Therapeutic effect of BMMCs	Spatial learning-memory ability of dementia rats was improved as demonstrated by by Morris water maze and the escape latency test.	The mechanism might be possibly correlated with mRNA expression level of NGF that was up-regulated in basal forebrain.	Zhongguo Zhong Xi Yi Jie He Za Zhi. 2011 Jun;31 [6]:799–802.
Yu, 2018	Random experiments, Sprague-Dawley female rats,in vivo study. Therapeutic effect of BMMCs	The expression of Seladin-1 and nestin were lower in the AD group when compared with the control group, whereas the BM-MSC transplantation reversed their down-regulation.	BM-MSC transplantation enhanced Seladin-1 and nestin expression potentially via a mechanism associated with the activation of the PI3K/Akt and ERK1/2 signaling pathways.	Oncol Lett. 2018 May;15 [5]:7443–7449.
Zhang, 2012	Sprague-Dawley rats,in vivo study. Therapeutic effect of BMMCs	BMMSCs plus BDNF resulted in significant attenuation of nerve cell damage in the hippocampal CA1 region. Tyrosine kinase B mRNA and protein levels were significantly increased, and learning and memory ability were significantly improved.	Increasing the levels of brain-derived neurotrophic factor and tyrosine kinase B in the hippocampus.	Neural Regen Res. 2012 Feb 5;7 [4]:245–50

**Table 2:** General characteristics of the included studies in this meta-analysis. Transplantation of BMMSCs for the treatment of animal models with Alzheimer’s disease was characterized by source of stem cells, amount of stem cells, animal species, gender, age, body weight, delivery method, etc.

Studies	Sources of transplanted stem cells	Amount of transplanted stem cells	Species of recipient animals	Gender ratio of recipients	Age or body weight	Route of delivery	Position of delivery	Sustainability of transplanted stem cells
Bae, 2013	Tibias and femurs were dissected from 4- to 6-week-old C57BL/6 mice	1 × 10^6^ of the cells in a 2uL volume	TASTPM mice (*n* = 9 for each group)	Female only	4 months of age.	Transplanted bilaterally into hippocampus	The following coordinates: 2 mm posterior to the bregma, 1.5 mm bilateral to the midline, and 2 mm ventral to the skull surface.	Mice were sacrificed at 2, 3, and 4 months after BMMSC transplantation.
Garcia, 2014	6-week-old C57BL/6-Tg (ACTBEGFP)10sb/J transgenic mice	1 × 10^6^ of the cells in a 5uL volume	2xTg-AD male congenic mice (APPswe/PS1dE9, B6.Cg-Tg (APPswe,PSEN1dE9)85Dbo/J)	Male congenic mice (*n* = 10/group)	6, 9 and 12 months of age	Lateral ventricle	The coordinates for stereotaxical injection (atlas by Paxinos and Franklin 2004) were used:−0.34 mm posterior to bregma, −0.9 mm lateral to the midline and 2.3 mm ventral to the skull surface	40 days after transplantation
Harach, 2017	Stem cells were obtained from Stemedica Cell Technologies (San Diego, USA). The cells are equivalent to commercially available stemcells from ThermoFisher Scientific “StemPro BM MSC” (part numberA15653).	5 × 10^5^ cells in 100uL of LRS	APP/PS1 mice	Male:female = 1:1 (*n* = 5/group)	1 ~ 12.5-month-old	Single intravenous or weekly intravenous for 10 weeks	Tail vein	10 weeks
Kanamaru, 2015	C57BL/6-Tg (CAG-EGFP)mice(4 weeks old, male)	5 × 10^6^ cells in 0.25 mL of HBSS	Tg2576 (APP) and DAL	Female only (*n* = 8 ~ 12/group)	6-month-old APP mice/9-month-old DAL mice	Peripheral vein	Retroorbital venous plexus	3 months/9 months
Lampron, 2013	Mouse femurs and tibias were dissected, and their bone marrow was flushed with phosphate-buffered saline (PBS) containing 5% fetal bovine serum, recipient mice were treated with a regimen of myeloablative chemotherapy prior to receiving bone marrow cells from GFP1 transgenic mice	2 × 10^7^	APP/PS1 and wild-type C57/BL6 mice	Unknown	7 ~ 8-week-old mice	Peripheral vein	Tail vein of recipient mice	2.5–10 weeks before they received any other treatment or surgeries.
Lee, 2010	4- to 6-week-old C57BL/6 mice	1 × 10^5^ cells in 3 μl of the cell suspension	Aβ induced AD (Aβ, *n* = 20; PBS, *n* = 10)	Unknown	4 ~ 6-week-old	Hippocampus bilaterally	The brain coordinates: 1.6 mm posterior to the bregma, 1.7 mm bilateral to the midline, and 1.2 mm ventral to the skull surface.	Mice were sacrificed at 11 days after BM-MSCs transplantation.
Lee, 2010	4 to 6-week-old C57BL/6 mice	1 × 10^4^ per mouse/3ul	APP/PS1 mice	Male mice	7 months 1 week of age	Hippocampus bilaterally	The following coordinates: 1.6 mm posterior to the bregma, 1.7 mm bilateral to the midline, and 1.2 mm ventral to the skull surface.	At 9 months of age, mice were killed and evaluated for changes.
Lee, 2012	Bone marrow of the mice expressing green fluorescent protein (GFP)	1 × 10^4^ per mouse/3ul	APP/PS1-GFP Chimeric Mice (*n* = 10 per group)	Unknown	7 months 2 week of age	Intracerebral hippocampus	The following coordinates: 1.6 mm posterior to the bregma, 1.7 mm bilateral to the midline, and 1.2 mm ventral to the skull surface	Mice were sacrificed at 3, 7, and 14 days after the last treatment.
Li, 2011	UBC-GFP mice with the genetic background of C57BL/ 6 J, UBC-GFP mice (8 to 10 weeks old)	1 × 10^7^ cells per mouse	APP/PS1 mice. Six weeks after bone marrow transplantation, mice were randomly divided into a saline control group (*n* = 5) and an SCF + G-CSF-treated group (*n* = 5).	Unknown	7-month-old APP/PS1 mice	Peripheral vein	Tail vein	After treatment for 9 months,the mice were sacrificed
Li, 2012	The 5th passaged human BMMSCs labeled with PKH26	1 × 10^6^ of the cells in a 1000uL volume	SD rats	Male only (10 rats per group)	3 months of age, ~ 300 g	Peripheral vein	Tail vein	14 days
Liu, 2015	Mouse BMMSCs overexpressed antisense of miRNA-937	1 × 10^6^ of the cells in a 5uL volume	APP/PS1 mice	Unknown; *n* = 10/group	9 months of age	Bilateral hippocampi	The stereotaxic coordinates were as follows: 2 mm posterior to the bregma, 2 mm bilateral from the midline, and 2 mm ventral to the skull surface.	At 9 month for SR and PM-DAT evaluation
Magga, 2012	Monocytic cells-derived from mouse or human bone marrow.	3 × 10^5^ in 1 ul of HBSS, 2% FBS	APPswe/PS1dE9 (APdE9) mice	Unknown, *n* = 5 for AD and *n* = 4 for WT mice	2-year-old	Intrahippocampal (right hippocampus)	The brain coordinates: 0.25 mm medial/lateral,0.27 mm anterior/posterior,0.25 mm dorsal/ventral from bregma.	After 4 days post-transplantation, the brains were collected
Matchynski-Franks, 2016	BMMSCs from C57BJL/6 or GFP-positive mice	2 × 10^5^ cells/μl in HBSS, 4 μl per mouse	5xFAD	Male:female = 1:1; LV (*n* = 8), [2] Hipp (*n* = 8), [3]LV-Hipp (*n* = 8), [4] WT Sham (*n* = 6), [5] AD Sham(*n* = 6),WT surgery control (*n* = 6), and AD surgery control (*n* = 6)	6 months of age	Central nervous system	Hippocampus and /or ventricle; A burr hole was drilled on each side of the skull, directly over the site of injection at −0.2 anterior/posterior from bregma (A/P) and ± 1.0 medial/lateral from bregma (M/L) into the ventricle, −1.2 A/P and ± 1.0 M/L into the hippocampus, or all four locations.	10 weeks after transplantation
Naaldijk, 2017	C57BL/6 mouse as a source for bone marrow-derived MSC. MSCs at passage 1–2 were used for transplantations	1 × 10^6^ of the cells in a 150uL volume	APP/PS1 mice	Male animal (day 7 *n* = 3 and day 28 *n* = 4), female recipients (day 28, *n* = 3), control mice *n* = 11	12 ~ 15 months of age	Peripheral vein	Tail vein	7 or 28 days animals were sacrificed
Ruzicka, 2016	Human mesenchymal stem cells (MSCs)	6 × 10^4^ cells/2 μLof saline	3xTg-AD mice. The 3xTg-AD mouse strain (LaFerla, Irvine, CA, USA), harboring three transgenes ofPS1 (M146V), tau (P301L) and APP (SWE), was used. Mice (saline-injected 3xTg-AD, *n* = 14; MSC-injected 3xTg-AD, *n* = 16; and WT controls without treatment, *n* = 14)	Unknown	8 months of age	left lateral ventricle	Coordinates from bregma: anteroposterior = 0 mm, mediolateraly = 1 mm, dorsoventraly = 2 mm	6 months
Safar, 2016	Bone marrow was aspirated from the femora and tibiae of adult male syngeneic Fisher-344 rats. The interphase layer containing bone marrowderived mononuclear cells (BM-MNCs) was collected, and the cells were washed twice with phosphate-buffered saline (PBS) before centrifugation at 400 g for 5 min.	2 × 10^6^ cells, BM-EPCs	Adult Wistar rats	Male only (12 rats per group)	Weighing 180–220 g	Peripheral vein	Tail vein	One month
Selem, 2014	Bone marrow was harvested by flushing the tibiae and femurs of 6-week-old male Sprague–Dawley rats with Dulbecco’s modified Eagle’s medium (DMEM, GIBCO/ BRL, Grand Island, NY, USA) supplemented with 10% fetal bovine serum (GIBCO/BRL).	3 × 10^6^cells/rat	Adult Sprague–Dawley rats, orally administered with aluminum chloride at 17 mg/kg b. wt. (Krasovskiietal.,1979) daily for75 days for induction of AD disease.	Adult female rats (8rats/group)	Weighing130–150 g	Intravenously	Tail vein in 5 min with a 27G needle	4 months
Wu, 2011	Bone marrow was harvested from Wister rat.	1 × 10^5^ cells in 5 μl/per side	SD rats	Male rats (15 rats per group)	3 ~ 4 months	Hippocampus bilaterally	Coordinates: 4.0 mm posterior to the bregma, 2.0 mm bilateral to the midline, and 3.0 mm below the dura mater.	One month
Yu, 2018	The femoral bones were harvested from 4 donor male rats.	3 × 10^6^ cells/rat in a single dose	Sprague-Dawley rat	Female rats (*n* = 8 per group)	Body weight 130-150 g	Peripheral vein	Tail vein	Unknown
Zhang, 2012	Six healthy Sprague-Dawley rats (used for cell culture), aged 2–3 weeks, weighing 80–120 g	5 × 10^6^ in 10 μl	A randomized, controlled, animal experiment. Adult Sprague-Dawley rats,	Male rats (??rats per group)	Weighing 280–300 g	Lateral ventricular	Stereotaxic Coordinates described by George Paxinos [4]: Neurobiol Aging. 2009;30 [3]:377–387; left ventricle was localized at 1.0 mm posterior to Bregma and 1.5 mm adjacent to the median, and 4.0 mm below the dura mater.	Tests were performed at 16 days and was completed at 20 days.

### Improvement of cognitive and behavioral deficits

**Fig. 3:**
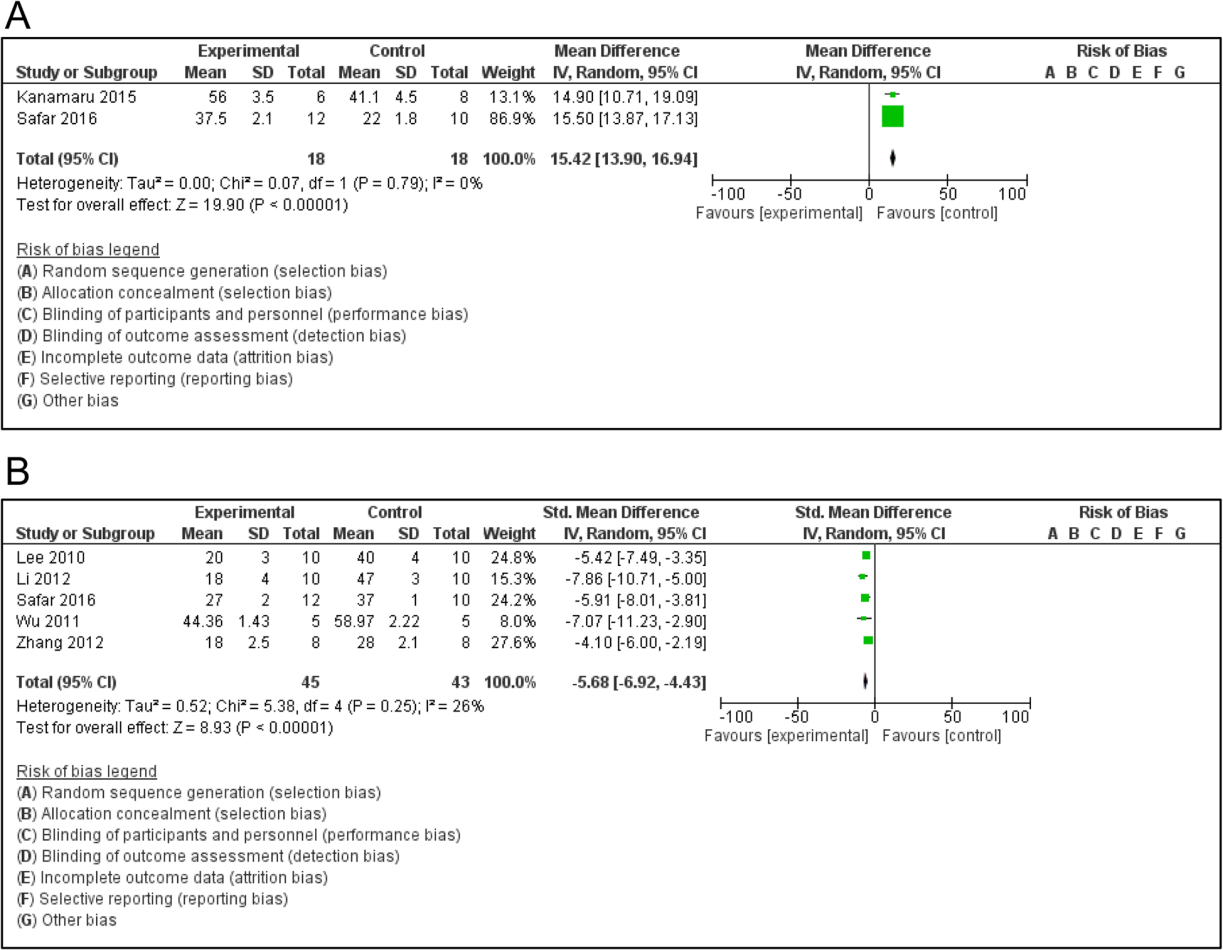
Transplantation of bone marrow mesenchymal stem cells could improve behavioral deficits in animal models of Alzheimer’s disease, which was generally characterized by abnormal manifestation or relationship. The beneficial change might be a temporary or permanent effect when compared to previous behavior. **a**. Behavioral changes as demonstrated through Y-maze test; **b**. Behavioral changes by Morris water maze test

The present review summarized therapeutic role of bone marrow mesenchymal stem cells in animal models of Alzheimer’s disease. The therapeutic effect of the bone marrow mesenchymal stem cells was demonstrated via behavioral changes in experimental subjects. After transplantation of bone marrow mesenchymal stem cells into Alzheimer-like animal models, symptom and sign were significantly alleviated as exhibited in APP mice, DAL mice or scopolamine-induced rats [12, 19, 20]. Benefits of the transplanted stem cells in the behavioral changes were confirmed through diverse tests such as Morris water maze test, Y-maze alternation test (Y-maze), plus-maze discriminative avoidance task, social recognition test and open-field evaluation (Fig. 3a, b). There was an improvement in learning ability and spatial memory performance subsequent to a transplantation of BMMSCs. The functional improvement of model brains was evidenced by preventive treatment against spatial learning and memory impairment. Of note, behavioral measurement was not performed in all experiments, because some animals were too young to conduct behavioral tests in certain studies [21].

Importantly, the BMMSCs treatment was beneficial in both young and aged Alzheimer-like animals. This therapeutic approach could reverse cognitive impairments induced by cerebral amyloidosis as observed in mouse AD models [3, 18, 21]. The treatment of transplanted BMMSCs could ameliorate spatial learning and memory impairment. Also, the BMMSCs treatment might improve impaired spatial memory in APP/PS1 mice as detected via Morris water maze test [3]. The APP/PS1 mice treated with BMMSCs had shorter escape latency than that of PBS-treated controls. These results indicated that the transplantation of BMMSCs was able to reduce the cognitive impairment of spatial memory [3]. Moreover, 3xTg-AD mice lost their working memory, but this impairment was improved in the transgenic mice after having received transplanted MSCs. The BMMSCs could dramatically alleviate working memory in the 3xTg-AD mice [22]. The transgenic DAL mice express a dominant-negative mutant form of mitochondrial aldehyde dehydrogenase 2 and exhibit AD-like phenotypes. By having employed a spontaneous Y-maze alternation test, an alternation rate of BMMSC-treated DAL mice was significantly higher than that of vehicle-treated mice in 3 months after transplantation. Even a single transplantation of stem cells was enough to have an effective result [12]. The cognitive decline could be ameliorated and even reversed via the beneficial role of BMMSCs in the AD animals [18].

Above-mentioned improvement was associated with input concentration of stem cells, cell viability, passage number, and delivery methods. The delivery routes of stem cells included (i) intravenous delivery. Animal models might receive either a single injection or a weekly injection more than 10 weeks through the tail vein [21]; (ii) intranasal administration of active factors secreted by stem cells. The animal was restrained by hand without anesthesia. An appropriate amount of soluble MSC factors was placed at nares of the animal via a pipette until the liquid drop disappeared into the nares [21]. A repeated intranasal delivery of soluble factors from cultured MSCs was enough to improve behavioral deficits in the mice; (iii) intracerebral or intracerebroventricular injection of stem cells. Intracerebral transplantation of grafted cells circumvents the prohibitive blood brain barrier and the cells can reach the discreet brain site. Benefits of mesenchymal stem cells on memory improvement in AD models had been detected [23]. However, the intracerebral delivery, compared to peripheral route, is an invasive procedure to implant stem cells into particular brain area [15]. Thus, it is a major hurdle for clinical applications. In contrast, intravenous delivery of transplanted stem cells is fast and easy route, and complications are rarely observed. To date, some preclinical studies have evaluated the impact of intravenous MSC injections on cerebral amyloidosis [21, 24].

### Neuropathological changes

#### Removal of Aβ plaques

**Fig. 4:**
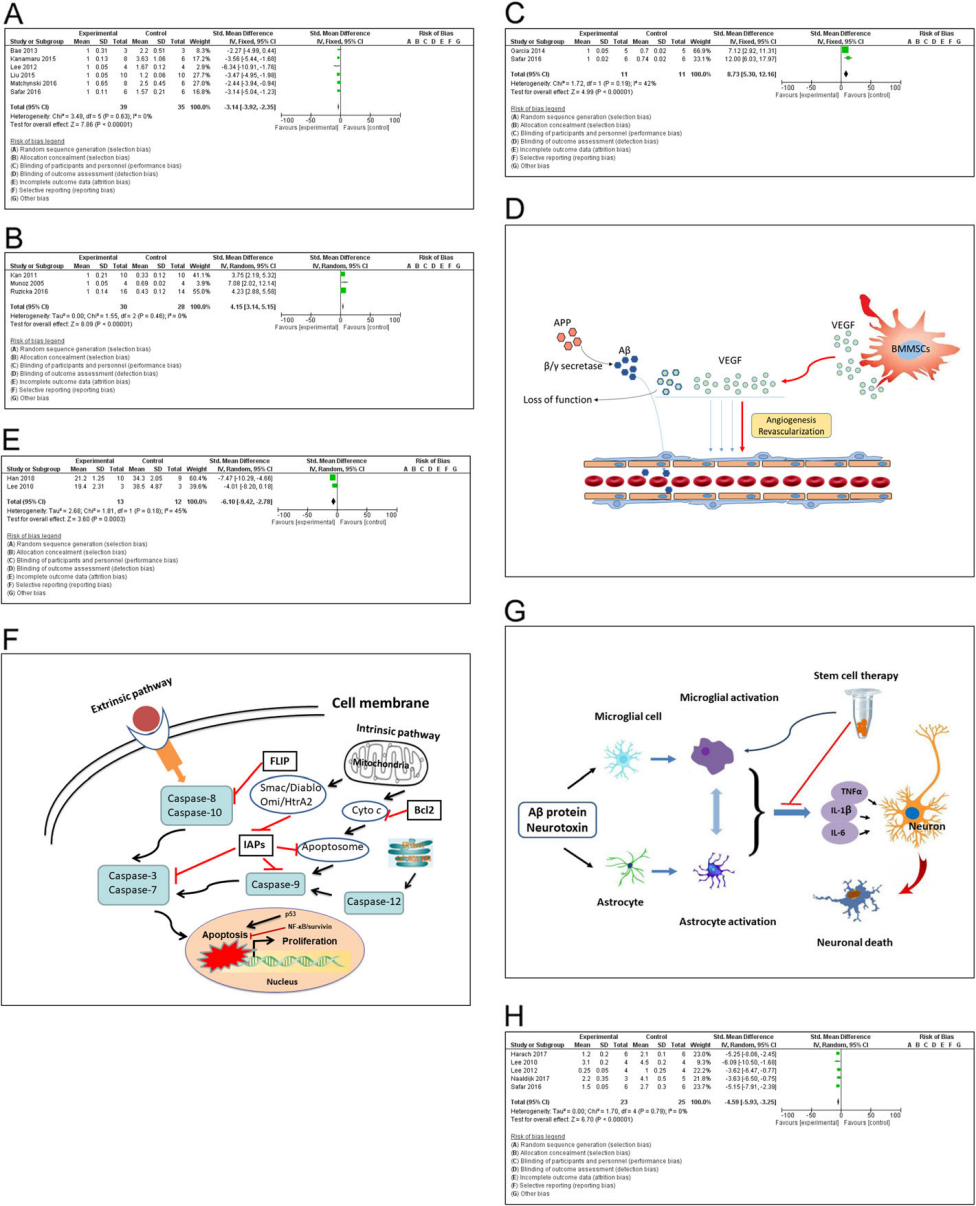
Meta-analysis on potential mechanisms. The transplantation of BMMSCs could alleviate neuropathology through diverse mechanisms, such as to decrease the number of hippocampal Aβ plaques as demonstrated in AD animal models (**a**). The Fig. 4a was plotted by relative ratio. The value in experimental group was assigned as 1 and the same as the following figures; to stimulate neurogenesis, neuronal differentiation, and neuronal integration (**b**); to promote angiogenesis in brain tissue as reflected by VEGF marker (**c, d**); to attenuate Aβ-induced apoptotic cell death in both primary hippocampal neurons and Aβ-injected animal models (**e, f**); immunomodulation and neuroprotection (**g**); to inhibit neuroinflammation in AD animal models (**h**)

Amyloid β peptide deposits in brain tissue and forms plaques. Moreover, the Aβ plaques are accumulated in special areas of AD brain. Nowadays cumulative level of Aβ plaques is a hallmark of AD. It is still a long way to demonstrate the actual role of Aβ plaques in the pathogenesis of Alzheimer’s disease, but the number of Aβ plaques is increased along with the deterioration of AD stage. The deposition of amyloid plaques in the form of spots and streaks could induce neuronal cell death via oxidative stress in the hippocampus [20, 25]. The transplantation of stem cells was able significantly to decrease the number of hippocampal Aβ plaques, which was demonstrated in APP/PS1 model mice as early as 1 week after intravenous delivery (Fig. 4a). Further investigation indicated that the impact of stem cells could activate several Aβ-degrading enzymes such as neprilysin-degrading enzyme, insulin degrading enzyme (I), endothelin-converting enzyme, etc. Those enzymes may play a critical role during degradation of amyloid β plaques. In the aspect of feasibility, the therapeutic application of stem cells via intravenous delivery is convenient and sufficient to diminish cerebral amyloidosis [21, 25].

#### Neurogenesis, differentiation and integration

The intravenous transplantation of stem cells was readily detected in brain parenchyma, i.e. hippocampus as revealed in 1 h after administration [21]. The expression of *sry* gene in the brain tissue of female AD model treated with male BMMSCs confirmed the migratory ability of the intravenously infused foreign stem cells to the site of brain injury [25]. The BMMSCs could differentiate into neuron-like cells and partially express ChAT [26]. Neural cells express nestin that can be as a marker of neural precursors. Brain nestin expression was up-regulated subsequent to the treatment of BMMSCs [27]. Bone marrow cells migrate throughout the brain and differentiate into neurons and glial cells [11]. In the hippocampus, there were different neurogenic phases such as proliferation, differentiation, migration, targeting, and integration respectively [28]. The transplanted stem cell may play a beneficial part in different phases of cell growth, although exact mechanism remains to be determined. The MSCs produce various trophic factors, including BDNF, NGF, and IGF-1 [29–31]. The MSCs could upregulate the trophic factors like NGF, FGF-2 and BDNF. This result could be attributed to the positive expression of growth factor, chemokine and extracellular matrix receptors on the surface of MSCs [25]. All these factors contribute to recover neurobehavioral function and stimulate endogenous regeneration. The BMMSCs could significantly increase the intensity of ChAT spots as well as the number of positive cells for ChAT expression in AD group. Cholinergic change is potential mechanism for the neurogenesis subsequent to a transplantation of BMMSCs. After BMMSCs treatment, the improvement in these biomarkers might be attributed to the powerful neurogenesis, neuronal differentiation and integration [11, 32] (Fig. 4b).

#### Angiogenesis

Angiogenesis is a pathophysiological process that is involved in regeneration and tissue reconstruction. Transplantation of the BMMSCs can promote angiogenesis in brain tissue as proved by (a) the fold change of expression marker such as VEGF; (b) interaction between VEGF and Aβ protein in experimental animal study; and (c) therapeutic effects of the VEGF in the murine model of Alzheimer’s disease [33–35]. The role of MSC in the cerebrovasculature had been correlated with angiogenesis and revascularization, mainly through secretion of various angiogenic factors (Fig. 4c, d). An administration of MSCs stimulated revascularization at the site of injury via secreting VEGF, FGF-2, Ang-1 and EGF [36]. The injection of hMSC into rats would increase angiogenesis by enhancing endogenous VEGF and VEGFR2 levels in the ischemic zone [37, 38]. Moreover, transplanted stem cells were able to differentiate into mural cells that accelerated the formation of peripheral vascular layers [39]. In the context of neurodegenerative disorders, these mesenchymal stem cells might contribute to neuroprotection by secreting trophic factors such as EGF, VEGF, FGF-2, NT-3, HGF, and BNDF [40]. Further study on potential mechanisms in AD models will be required to understand the contribution of above factors to the disruption of amyloid plaques following intravenous implementation of stem cells [21]. In the brains of AD patients, the soluble VEGF concentration is decreased because Aβ binds to VEGF forming aggregate that leads to the loss of angiogenic and neuroprotective activities [41]. Therefore, provide additional VEGF would have high therapeutic effect. An overexpressing VEGF in mesenchymal stem cells could promote neovascularization in the hippocampus and recovered the memory deficit in the 2xTg-AD animals. More interestingly, only intraperitoneal injection of VEGF could improve cognitive function through the hippocampal angiogenesis and decreased Aβ deposition in the brain [35, 42].

#### Inhibition of apoptosis

The Aβ peptide in AD animal models could induce neuronal apoptosis via caspase pathway [13, 43, 44]. The neuronal apoptosis was responsible for the memory impairment in AD brain. The transplantation of BMMSCs attenuated Aβ-induced apoptotic cell death in primary hippocampal neurons as well as intrahippocampally Aβ-injected AD animal models (Fig. 4e, f). The neuroprotective mechanisms of BMMSCs may be through (a) to reduce Aβ deposition. The Aβ peptide induced the stress-activated protein kinases p38 and c-jun N-terminal kinase, and upregulated p53 expression, which were closely associated with apoptosis [1]. Furthermore, the MSCs expressed seladin-1, which inhibits the activation of caspase-3 and is a neuroprotective factor. The transplantation of BMMSCs could significantly increase seladin-1 gene expression in AD groups [45]; (b) activation of the cell survival signal pathway. The BMMSCs treatment upregulated the survivin expression as showed by the increased number of survivin-positive cells in AD models [46]. The MSCs could inhibit P53 activation [47]. Also, the MSCs produce VEGF, BDNF, NGF, and FGF2, which were supposed to exert an anti-apoptotic effect [48]. The BMMSCs could significantly down-regulate caspase-3 expression, thus protecting seladin-1 from cleavage [49]; (c) to decrease oxidative stress-induced neurotoxicity in the hippocampus [18]. ER-oxidative stress and mitochondrial failure involve the pathogenesis of Alzheimer’s disease. The transplantation of stem cells led to a significant improvement of memory deficits in AD mouse models via the suppression of apoptosis and the maintenance of functional synaptic contacts [4, 13, 50]. The MSCs could up-regulate the cellular antioxidant defense through their capability to secrete trophic factors like NGF, FGF2 and BDNF. MSCs could also attenuate oxidative damage by reducing ROS and increasing expression of endogenous antioxidants in neurons [47]. The apoptotic mechanism not only took part in neuronal cell death, but also involved survival of transplanted mesenchymal stem cells in brain tissue. Actually, the later also hampered the clinical application of stem cell therapy for Alzheimer disease [51].

#### Immunomodulation

Histopathological examination disclosed that immunomodulatory property of the BMMSCs play an important role in therapeutic role against AD as well [25]. The intracerebral transplantation of BMMSCs was applied to acute AD model induced through Aβ peptide injection into the dentate gyrus of hippocampus of C57BL/6 mice. The activation of microglia promoted the diminution of Aβ deposits due to microglial phagocytosis. The BMMSCs could accelerate the activation of microglia and the removal of Aβ deposition in AD brain [52]. In vitro study demonstrated the bone marrow-derived mesenchymal stem cells could decrease expressional levels of pro-inflammatory genes (IL-1β, TNF-α, IL-6) in astrocytes [53]. The MSCs regulated a series of gene expression, including intermediate filaments (GFAP, vimentin), pro-inflammatory enzymes (iNOS, COX-2) and receptors (TLR4, CD14, mGluR3, mGluR5). Immunomodulatory influence of MSCs may be through diverse cell types to participate in the neuroinflammation (Fig. 4g). The observation of decreased neuroinflammation in hMSC-treated APP/PS1 mice further suggests that hMSC delivery does not elicit a major immune response from the host. In addition, preclinical study demonstrated that a repeated intravenous hMSC treatment could safely reduce cerebral Aβ pathology in a typical mouse model of AD.

#### Inhibition of inflammation

The neuroinflammation was reduced in APP/PS1 mice following hMSC treatment [21] (Fig. 4h). There was a dramatic decline on the panel of cerebral cytokines such as IFNγ, diverse interleukins (IL-1β, IL-2, IL-4, IL-5, IL-6, IL-10, and IL-12p70), KC/GRO, and TNF-α, suggesting an anti-inflammatory impact of hMSCs. The hMSC treatment significantly down-regulated cerebral IBA-1. Among multiple cell types of brain tissue, the IBA-1 gene is specifically expressed in microglia. Upon activation of microglia due to inflammation, expression of the IBA-1 is up-regulated, which allows the discrimination between surveilling and activated microglia. Microglial coverage was examined to evaluate neuroinflammation changes in transgenic brains following repeated hMSC treatment [21]. There was an overall decrease of the microglia coverage in brains of APP/PS1 transgenic mice of both young and aged groups. A qualitative observation was confirmed by quantitative image analysis of IBA-1 immunoreactivity. TNFα and IL-12p70 were reduced following a single hMSC intravenous injection. Interestingly, TNFα has been implicated in chronic inflammation, cancer, and other inflammatory diseases. Notably, levels of the cytokine IL-10 were decreased following stem cell treatment, which might be therapeutically relevant for AD although this cytokine was reported to be anti-inflammatory. Accordingly, AD patients showed abnormally high IL-10 signaling, which highlighted that blocking the IL-10 anti-inflammatory response could be therapeutically relevant for AD [54]. The repeated intravenous hMSC injections or even single administration reduced cerebral neuroinflammation. The anti-inflammatory role of BMMSCs was also verified in a rat model of spinal cord injury [55]. Obviously, the stem cell therapy significantly inhibited the inflammatory response.

### Gene-specific patterns of Alzheimer’s disease

**Fig. 5:**
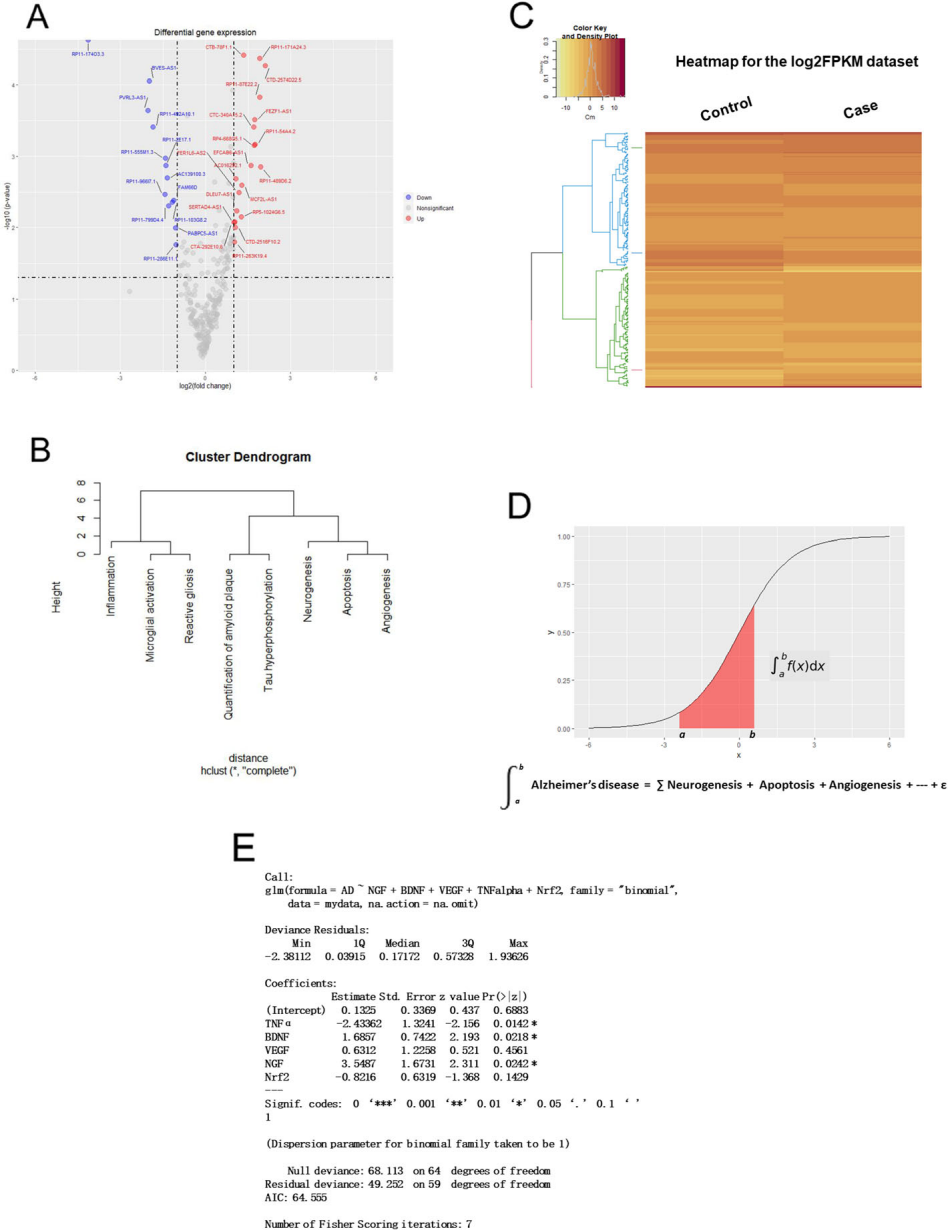
Construction of gene-specific regression model. **a**. Differential gene expression was compared between control and samples of patients with Alzheimer’s disease. **b**. Hierarchical cluster analysis based on the comparison between control and gene data from samples of patients with Alzheimer’s disease. **c**. Heatmap of gene data from brain samples of patients with Alzheimer’s disease. **d**. Sigmoid curve of gene pattern. **e**. Logistic regression equation for prediction of gene-specific patterns of Alzheimer’s disease

In pathology, the pathogenesis of Alzheimer’s disease can be classified into different stages, which involves various mechanisms such as proliferation, apoptosis, angiogenesis, immunomodulation, inflammation, etc. These mechanisms are reflected by differential gene levels as compared with normal control (Fig. 5a). In recent decades, gene analysis based on microarray assay and high-throughput DNA sequencing has provided abundant information on gene expression profile of Alzheimer’s disease. It is reasonable to hypothesize that the Alzheimer’s disease has gene-specific patterns by which its progression and severity are mediated.

Gene data from microarray assay and high-throughput DNA sequencing were collected and analyzed through comprehensive comparison. In gene ontology and signal pathway analyses, principal components of differential genes were identified [56]. The guideline for the construction of gene-specific patterns was summarized as follows:

1. Comparison of differential gene expression in brain samples of patients with AD (Fig. 5b).
  Quantification of hippocampal key genes, such as BDNF, NGF, VEGF, etc.
  Estimation of inflammatory cytokines, such as TNF-α, IL-1β, IL-10, etc.
  Determination of oxidative damage, e.g., hippocampal Nrf2 level.
2. Cluster analysis of all relevant gene data (Fig. 5c).
3. To screen principal variables via PCA analysis.
4. Statistical regression model. After correlation and regression analysis, a multinomial logistic equation was obtained (Fig. 5d, e).
  Based on big data analysis, a predictive model was composed of representative gene variables in the pathogenesis of Alzheimer’s disease.
  Logistic regression equation can classify gene variables into gene-specific patterns.
5. Pathophysiological significance of the gene-specific patterns.
  To diagnose patient based on differential gene levels. Logistic regression model can distinguish AD patient from normal control.
  To predict progression of AD, severity, and patient’s life expectancy.

In the context of neurodegenerative AD, the transplantation of BMMSCs could improve cognitive deficits and alleviated neuropathology at various degrees. The grafted MSCs contributed to neuroprotection through secretion of neurotrophic factors such as BDNF, EGF, VEGF, FGF-2, NT-3, HGF and so forth [40]. Differential gene expression involved a series of functional results of paracrine secretion of neurotrophic factors and cytokines. The aforementioned changes might be weighed by differential levels of responsible genes. In fact, therapeutic effect of the BMMSCs was determined by comprehensive role of representative genes. As presented in this study, there are gene-specific patterns in the pathogenesis of Alzheimer’s disease. The gene patterns would be an appropriate method to assess the therapeutic effect subsequent to stem cell transplantation in AD models. Accordingly, relative levels of representative genes can be used to evaluate the progress and prognosis of the disease. Next, it is necessary to expand the sample size of representative gene data and further to confirm real contribution of these key genes to the pathogenesis of Alzheimer’s disease.

## Discussion

Therapeutic effect of the transplanted BMMSCs was demonstrated with the improvement of memory loss and behavioral deficits in animal models with Alzheimer’s disease [18, 57, 58]. Positive results have been acquired not only through the repeated transplantation of BMMSCs, but also via a single injection or even soluble MSC factors over nasal mucosa. In future, it is possible to use BMMSCs for the clinical treatment of Alzheimer’s disease. Potential mechanisms are associated with a broad coverage of neurogenesis, differentiation, apoptosis, angiogenesis, inflammation, immunomodulation and so on [17, 18, 20, 22]. However, the exact mechanism remains to be determined. Based on data analysis, a gene-specific pattern was revealed in brain tissue of patients with Alzheimer’s disease. The above gene patterns were altered with the severity of neuropathology, which maybe a useful tool for the molecular diagnosis and therapeutic evaluation of Alzheimer’s disease.

It is a long way to clarify the pathogenesis of Alzheimer’s disease. However, an investigation on its potential mechanisms is still an essential work, since any progress in clinical treatment depends on a comprehensive understanding of the relevant mechanisms. Neuropathological mechanism is associated with differential panel of gene expression [21, 54, 56]. Gene change in brain tissue can be clustered into diverse patterns based on expressional levels and functional features. Therefore, a novel concept of the gene pattern is proposed. The gene pattern may be utilized as a surveillance marker for the dynamic assessment of neuropathology. Its significance will be reflected in the molecular diagnosis and therapeutic evaluation of Alzheimer’s disease.

Beneficial results of BMMSCs transplantation had been observed in different animal models that were induced using genetic modification, Aβ protein injection, or administration of chemicals. The transplantation of stem cells from autologous BMMSCs did not cause any immune response. Enormous experiment data showed therapeutic effects of the BMMSCs, which included the improvement in cognitive deficits and pathological changes [18, 20]. It is quite possible for the BMMSCs to be utilized in clinical treatment of AD patients in future, because (a) stem cells are easily obtained through bone marrow aspiration; (b) peripheral vein delivery; (c) autologous stem cells without immunogenicity.

A combination of transplanted BMMSCs with drug therapy may be a future direction. In clinical, cholinesterase inhibitors and NMDA antagonist have been now used to improve memory loss and behavioral symptom of patients with Alzheimer’s disease [5, 6]. Therapeutic effect had been observed in certain patients, but not all patient community. If above-mentioned medications are combined with a transplantation of BMMSCs, what will happen? So far, it is only a rational hypothesis. In addition, the soluble factors from stem cells could also produce positive result, which encourages further investigation using the combination of neurotransmitter drugs with cytokines [21]. Their joint application may trigger a synergistic effect.

It seems that the stem cells from autologous bone marrow have some advantages as compared with those from allogeneic embryos and umbilical cord. However, there is still weakness in the transplantation of BMMSCs. There are some side-effects from bone marrow aspiration. Another drawback is from the preparation of stem cells. Moreover, there are diverse subtypes of stem cells according to CD markers on the cell membrane [59]. They can be also classified into disparate subgroups. Different cell subtypes may play distinct roles during neurogenesis and functional reconstruction. Unfortunately, it is remains to be identified for specific subtypes to give rise to precise roles and neuroprotective mechanisms.

## Conclusion

In summary, the beneficial effect was confirmed in animal models with Alzheimer’s disease subsequent to the transplantation of bone marrow mesenchymal stem cells. The therapeutic efficacy and safety were verified through the improvement of behavioral deficits and the alleviation of neuropathology. Multiple signal pathways involved therapeutic mechanisms, including neurogenesis, apoptosis, angiogenesis, immunomodulation, inflammation and so on. Gene expression profiles might reflect relative importance of above mechanisms in different stages. The transplantation of BMMSCs could alter gene expression levels. Differential expression of representative genes could be used to establish statistical regression model for the evaluation of therapeutic effect and the prediction of prognosis. There is a great possibility for the clinical application of autologous BMMSCs in patients with Alzheimer’s disease.

## Supplementary information

**Additional file 1: Table 1.** Stem cell transplantation for the treatment of Alzheimer’ disease. Present study utilized keywords “Alzheimer’s disease” and “stem cell transplantation” to identify literature. The supplementary table further scrutinized relevant information of stem cell transplantation in different animal models.

## Data Availability

All generated or analyzed data are included in this published article.

## Abbreviation

AD: Alzheimer’s disease
BMMSCs: Bone marrow mesenchymal stem cells
Aβ: Amyloid β peptide
AChE: Cholinesterase
NMDA: N-methyl-D-aspartic acid
iPSc: Induced pluripotent stem cell
MSCs: Mesenchymal stem cells
VEGF: Vascular endothelial growth factor
TNF-α: Tumor necrosis factor alpha
IL-1β,: Interleukin 1 beta
ECE: Endothelin converting enzyme
Iba-1: Induction of brown adipocytes 1
AT8: ATPase subunit 8
APP: Amyloid beta precursor protein
Y-maze: Y-maze alternation test
APP/PS1: Amyloid beta precursor protein/ presenilin 1
3xTg-AD: APP/PS1/Tau transgenic AD
ChAT: Choline acetyltransferase
BDNF: Brain-derived neurotrophic factors
NGF: Nerve growth factor
IGF-1: Insulin-like growth factor-1
FGF-2: Fibroblast growth factor 2
Ang-1: Angiopoietin 1
EGF: Epidermal growth factor
hMSC: Human mesenchymal stem cells
VEGFR2,: Vascular endothelial growth factor receptor 2
NT-3: Neurotrophin-3
HGF: Hepatocyte growth factor
2xTg-AD,: APP/PS1 transgenic AD
p38: P38 kinase
p53: Tumor protein p53
ER: Endoplasmic reticulum
ROS: Reactive oxygen species
IL-6: Interleukin 6
GFAP: Glial fibrillary acidic protein
iNOS: Inducible nitric oxide synthase
COX-2: Prostaglandin-endoperoxide synthase 2
TLR4: Toll like receptor 4
CD14: CD14 molecule
mGluR3: Metabotropic glutamate receptor 3
mGluR5: Metabotropic glutamate receptor 5
IFNγ: Interferon gamma
IL-2: Interleukin 2
IL-4: Interleukin 4
IL-5: Interleukin 5
IL-10: Interleukin 10
KC/GRO: Cxcl1 chemokine (C-X-C motif) ligand 1
ChIP: Chromatin immunoprecipitation
Nrf2: Nuclear factor erythroid 2-related factor 2
PCA: Principle component analysis
